# Evaluation methods for detecting changes in beam output and energy in radiation beams from high-energy linear accelerators

**DOI:** 10.4103/0971-6203.35720

**Published:** 2007

**Authors:** R. Ravichandran, J. P. Binukumar, C. A. Davis, K. Krishnamurthy, S. S. Sivakumar

**Affiliations:** Medical Physics Unit, Department of Radiation Oncology, National Oncology Center, The Royal Hospital, Oman

**Keywords:** Calibration of linacs, perspex phantoms, quality assurance methods

## Abstract

There is need for simple methods for checking consistency of beam outputs and energy in linear accelerators used for radiotherapy. A method was designed by the department using perspex phantom with which the dosimetric data of two medical linear accelerators (Clinac 600 CD, Clinac 2300 CD) were evaluated over a period of 30 months. The efficacy of methods followed was checked. Routine beam consistency checks were designed for photon beams with 15 cm/ 5 cm depth ionizations in perspex phantom and variable depth combinations for electron beams. Calculated ionization ratios were compared with measured values to show their significance. The dose/MU for all radiation beams was maintained within 2% accuracy over the period of 30 months. Clinac 600 CD machine showed decreasing trend of cGy/MU, while Clinac 2300 CD showed increasing trend of cGy/MU over a period, which needed tuning of monitor chamber two times each. Tuning of output to achieve standard value was carried out once, for all electron energies when the output dose/MU exceeded 3%. During one week (June 2005), there were slight changes in electron energy detected using the ratio method, which did not recur anytime afterwards. The methods designed are adequate to find the consistency in the beam output and energies in the radiotherapy linacs.

For clinical use of the linear accelerator beams, viz., photons and electrons, for patient treatments, the accuracy in delivery of absorbed dose should be within ±5%. With the advent of special techniques of dose delivery, such as intensity-modulated radiotherapy (IMRT), standards of dosimetry need to be more stringent for correct dose delivery with execution of static and dynamic radiotherapy fields.

Water or solid phantom measurements with appropriate dosimetry protocols (NACP1980, HPA 1983, AAPM 1983, IAEA 1987, IAEA 1997, AAPM 1999, IAEA 2000) are used to determine the physical parameters of megavoltage photons and electron beams.[1–7] To detect any change in photons and electron beam output, we need an in-phantom ionization measurement in reproducible geometry and field conditions, over a period of time, and comparison with the benchmark value. There is need for standard methods to verify the dose output and penetration of the beams remains constant. Any change in output dose/MU will reflect variation in response to transmission monitor chamber. When there are changes in absorbed doses showing systematic shift more than 2% detected by daily and weekly quality assurance measurements, changes are made in the treatment planning systems (TPS) for dose calculation purposes. Otherwise, the monitoring chamber sensitivity is adjusted to restore to standard outputs calibrated at the time of commissioning of the linear accelerators. Therefore, periodic quality assurance (QA) of the outputs and beam qualities is recommended for safe use of linear accelerators for radiotherapy. However, there are no uniform methods followed in different centers. At our center we have developed simple methods for QA using perspex phantom, and the performance of the linacs are highlighted.

## Materials and Methods

### Treatment machines

Measurements have been performed for a period of 30 months on the following two linear accelerators: 1) Clinac 2300 CD (M/s Varian AG, USA) with dual x-ray energies 6 and 15 MV and electron energies 6, 9, 12, 15, 18 and 22 MeV. 2) Clinac 600 CD (M/s Varian AG, USA) with 6 MV photons. The beam qualities of photons, TPR 20/10 are 0.6718 and 0.7608 for 6 MV and 15 MV respectively. The 6 MV beams have the same beam quality in both linacs.

### Consistency checks of beam output dose/MU and beam quality

#### Photons:

The dose outputs and energy consistency of photon beams were checked every week using commercially available 30 cm × 30 cm lucite phantom sheets and FC 65 chamber. Two measurements at 95 cm focus phantom surface distance (FSD) with variable depths of chamber at 5 cm and 15 cm depths were performed, keeping constant the total thickness of the phantom at 20 cm. First (5 cm) depth corresponds to ‘isocenter’ at 100 cm focus chamber distance (FCD). The readings obtained at 5 cm depth corrected for ambient conditions of measurements were taken to represent prevailing relative output of the machine.

**Figure 1:**
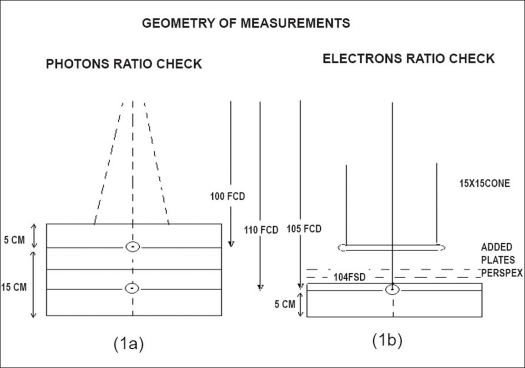
Geometry of perspex phantom measurements for weekly check of photon dose output, photon beam energy (a), electron beam dose output and beam energy (b). The chamber location and thickness details are seen

The ratio of ionization readings measured at 15 and 5 cm is taken to represent the effective penetration of the beams, and therefore the photon energies qualitatively. The geometry of chamber position and relative depths in the central axis of the beam are shown [Figure 1a]. Whenever the output drift observed was beyond 2%, tuning of dose/MU was carried out to restore the output back to reference dose/MU obtained during commissioning of the linacs.

#### Electrons:

The total thickness of the lucite phantom for QA check of the electron beams is variable, with FCD 105 cm. The first set of measurements was carried out at depth 10 mm [Figure 1b] with 50 mm backscattering perspex thickness. The ionization readings with 15 cm × 15 cm cone were noted for all the six electron energies. Perspex plates measuring 10 mm were later added for each step of increasing electron energy, maintaining the same 105 cm FCD geometry. The ratios of ionization with ion chamber at 10 mm depth to ionization with ion chamber at different other depths, viz., 20 mm, 30 mm, 40 mm, 50 mm, 60 mm, 70 mm, were correlated for the six electron energies 6, 9, 12, 15, 18, 22 MeV respectively. The above measurements were carried out weekly. The 10-mm readings corrected for ambient conditions were taken to represent output and the ratios to represent the penetration curves of the electron beams.

### Calculation of ratios of ionizations in phantom for photons and electrons

Ratios of ionizations were referred to water ionization curves for 6 MV and 15 MV photons and calculated for ‘15 cm and 5 cm’ and ‘17.75 cm and 5.95 cm’ (water equivalent mg/cm^2^) combinations. Means of these ratios were also calculated. For electron beams the ratios of ionizations at 20/10, 30/10, 40/10, 50/10, 60/10, 70/10 depth combinations were calculated. Depths of water were directly considered instead of water equivalence of perspex. The calculated ratios were compared with measured ratios at corresponding depth combinations.

### Performance of beam level dosimeters

The correctness in functioning of field dosimeters (used for the solid phantom measurements) was checked against departmental reference dosimeter and with a Strontium-90 check source (Scanditronix Wellhofer, Germany). Table 1 shows the stability of calibration of the dosimeters used for beam checks vis-á-vis departmental reference dosimeter.

**Table 1:** Comparison of response of beam level dosimeters with reference instrument

*Measured during month*	*Phantom*	*Reference dosimeter machine/beam*	*Measured response dosimeter 1 750/8740*	*Measured response dosimeter 2 750/8736*
		839/8763		
July 2005	Perspex	600CD 6MV	1.0090	—
		2300CD 6MV	1.0020	1.0016
		2300CD 15MV	1.0020	1.0000
Dec 2005	Water	2300CD 6MV	1.0050	1.0008
		854/8763		
Mar 2006	S.Water	2300CD 6MV	0.9968	0.9963
		2300CD 15MV	0.9967	0.9969
Dec 2006	S.Water	600CD 6MV	1.0050	1.0030
		600CD 6MV	1.0050	1.0022

## Results

### Measured outputs

#### Photons and electrons:

**Figure 2:**
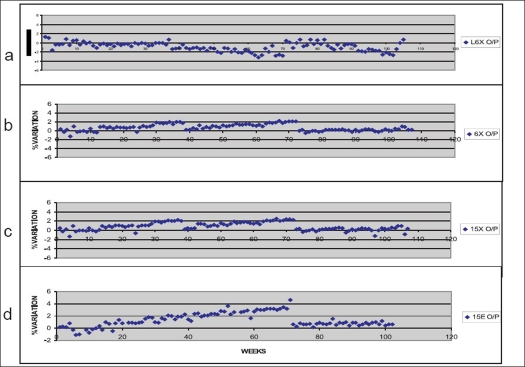
Measured percentage variations in dose output measured in perspex phantoms for a) 6 MV X-rays from Clinac 600 CD b) 6 MV X-rays from Clinac 2300 CD c) 15 MV X-rays from Clinac 2300 CD and d) 15 MeV electrons from Clinac 2300 CD

Relative percentage variations in dose output (for photons) during the period September 2004 to January 2007 (30 months) is shown in Figure 2. It can be seen that for Clinac 600 CD machine, there was a trend of drop in output/MU; and for Clinac 2300 CD machine, there was a trend of increase in output/MU over periods, which needed re-tuning. Change of dose/MU by tuning the monitor chamber response was carried out two times each for photon beams in both the linacs (April, December 2006 for Clinac 600 CD; July 2005, May 2006 for Clinac 2300 CD). Figure 2 (electrons) shows the trend observed in variation of output/MU in Clinac 2300 CD for 15 MeV electrons. All the other electron energies showed similar trend in the direction of positive percentage deviation in output. Only once, the dose/MU tuning was carried out for all the six electron energies.

### Measured ratios in perspex

**Figure 3:**
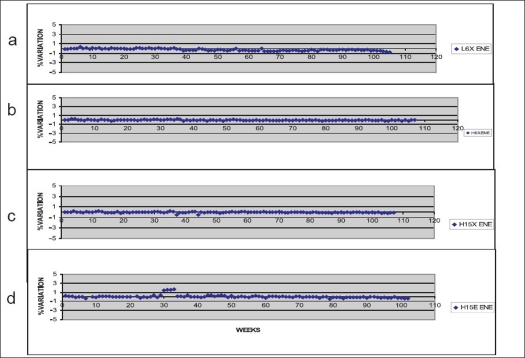
Measured percentage variations in measured ratios of ionizations at two reference depths in perspex phantom a) 6 MV X-rays from Clinac 600 CD b) 6 MV X-rays from Clinac 2300 CD c) 15 MV X-rays from Clinac 2300 CD and d) 15 MeV electrons from Clinac 2300 CD

**Figure 4:**
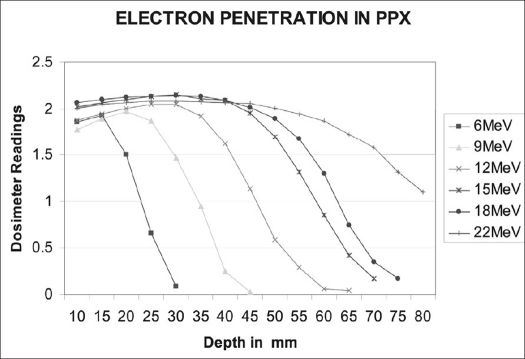
Relative dosimeter readings for various electron energies in perspex for different overlying perspex thickness above chamber. FCD 105 cm is maintained constant

**Figure 5:**
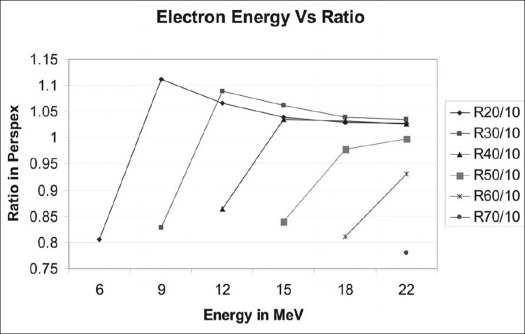
Cross plot of ratios of ionizations from measurements of Figure 4 shown against corresponding electron energies. Straight line portion of the ratio curves for different depth combinations corresponds to rapid fall of region of ionization curves of Figure 4

Tables 2 and 3 show that calculated and measured ratios for different depth combinations in photon and electron beams were in good agreement within 2% (for photons) and 3% (for electrons). The measured photon ratios in both machines remained constant within 0.5% [Figure 3] over a period of 30 months, confirming correct beam quality. Clinac 600 CD linac, however, showed more spread in the measured ratios compared to the same 6 MV beam from Clinac 2300 CD. The ratios for all the electron energies were constant within 0.5% over this period [Figure 3]. It can be seen that during one week (May 31 to June 7, 2005), we observed change in these measured ratios in perspex for all electron energies more than these limits (with statistical significance *P* < 0.001) [Table 4]. Figure 4 shows the relative ionization readings for various depths of perspex for different electron energies. The variations of ratios of ionizations for different depth combinations at different electron energies are shown in Figure 5. A difference of 0.2 MeV in nominal energies was predicted when there was change in ratios (June 2005) using Figure 5. The blue phantom measurements confirmed these variations in nominal electron energies [Table 5].

## Discussion

The present work has highlighted the results of QA measurements and their efficacy in evaluating the photon and electrons beam outputs and energy stability. The radiation field analyzer flatness and symmetry measurements; and weekly XLite phosphorescent screen measurements, isocentric cylindrical tool measurements during the past ensured the correctness of other field parameters. The methods suggested in this report take less time in the accelerator. Seuntjens *et al.*[8] indicated the corrections required to derive the absorbed dose from the readings at specified depths in solid phantoms such as solid water and Lucite. With these corrections, our measured perspex readings could be converted to absorbed dose in water for comparing with true output of the beams.

Most of the radiotherapy centers use lucite phantom sheets for checking ratio of penetrations with 20/ 10 cm depth ionizations. Because of density and effective atomic number differences, these measurements do not provide TPR 20/10 ratios. There are no reports on the numerical variations in the ratios with lucite correlating to the photon energy spectrum, because the attenuation and scattering properties of perspex are different from water (ρ_perspex_ = 1.19; z_eff_ = 5.85; H: C: O = 8%: 60%: 32%). Therefore, deriving an explanation for measured ratio of ionizations at different depths for photons becomes difficult. For indirect measurements of electron energies with solid phantoms, presently there are no recommended combinations of depths.

For photons, instead of 20/10 cm combination, we have selected 5 and 15 cm depths in perspex phantom for the following reasons. The 5 cm measurement could provide information on the stability of photon beam output because this depth is beyond depth of ‘dose maximum’ for both 6 MV and 15 MV photons. Assuming 5 cm depth backscattering and 5 cm depth forward scattering sufficient for 6 MV and 15 MV photons, a total thickness of phantom 20 cm is selected for this study. Ratios of ionizations were calculated for ‘15 cm and 5 cm’ and ‘17.75 cm and 5.95 cm’ (water equivalent mg/cm^2^) combinations for 6 MV and 15 MV photon energies. Only physical depths were considered, ignoring inverse square law. The calculated means of these two ratios are in agreement with the measured 15 cm/ 5 cm ratios [Table 2]. This may imply that the extra attenuation in perspex is compensated by the extra scattering for these photon beams in perspex medium. Our preliminary results were communicated earlier.[9]

**Table 2:** Comparison of measured and calculated ratios of ionizations at 15 cm/5 cm depths

*Photon energy*	*Measured ratio 15/5 perspex*	*Measured ratio 15/5 SW (SP34)*	*Calculated ratio 15/5 from water depth dose (A)*	*Calculated equivalent 17.75/5.75 water depths (B)*	*Calculated mean ratio (A+B)/2*	*Measured and calculated mean ratio % deviation*
6 MV	0.5460	0.5731	0.5879	0.5060	0.5469	−0.2
15 MV	0.6220	0.6442	0.6526	0.5714	0.6120	+1.6

For electron beams, different combinations of depths were selected for checking beam quality. A 10 mm first depth is near the depth of ‘dose maximum’ for all these electron energies. Other depths, 20 mm to 70 mm, are selected because these depths are in the rapid fall-off regions of the respective percentage depth dose curves in water. No fixed combinations of two depths could be arrived at to represent all the energies, because of the shapes of ionization curves in perspex for different energies [Figure 4]. In case of electrons when we take ratios of ionizations in water for corresponding depth combinations in perspex (assuming same mg/cm^2^ as water), the calculated ratios agree with measured ratios within 2.5% [Table 3]. This may mean that there is excess scattering at the reduced depths, making the ratio equivalent to that of water.

**Table 3:** Comparison of measured and calculated ratios of ionizations for electrons

*Electron energy*	*Depth combinations in perspex*	*Measured ratios for depth combinations (A)*	*Calculated ratios for same water depths (B)*	*Deviation measured and calculated ratios (%)*
6 MeV	20/10	0.8062	0.8163	−1.3
9 MeV	30/10	0.8293	0.8511	−2.6
12 MeV	40/10	0.8632	0.8842	−2.4
15 MeV	50/10	0.8409	0.8484	−0.9
18 MeV	60/10	0.8149	0.8300	−1.9
22 MeV	70/10	0.7887	0.7939	−0.7

We have brought out the specificity of these measured ratios in electron beam representing the nominal energy. It can be seen from Figure 5 that there is steep variation in these ratios with change in incident electron energies and we are able to predict changes as low as 0.1 MeV. With the changed ratios [Table 4], about 0.2 MeV variations in electron energies were predicted. The RFA measurements also confirmed the change in energies during the particular week [Table 5].

**Table 4:** Changed values of ratios in perspex for electron energies

*Energy*	*Depth combinations*	*Standard ratios*	*Changed ratios*	*P value*
6 MeV	20/10	0.8078 ± 0.00154 (n=98)	0.8368 ± 0.00573 (n=6)	<0.001
9 MeV	30/10	0.8291 ± 0.00142 (n=98)	0.8515 ± 0.00428 (n=6)	<0.001
12 MeV	40/10	0.8624 ± 0.00117 (n=98)	0.8779 ± 0.00295 (n=6)	<0.001
15 MeV	50/10	0.8394 ± 0.00125 (n=98)	0.8514 ± 0.00234 (n=6)	<0.001
18 MeV	60/10	0.8136 ± 0.00095 (n=98)	0.8228 ± 0.00018 (n=6)	<0.001
22 MeV	70/10	0.7874 ± 0.00113 (n=98)	0.7948 ± 0.00220 (n=6)	<0.001

**Table 5:** Comparison of electron energies after changes observed in ratios of ionizations

*Electron energies*	*RFA measurements Aug-Sept 2004*				*RFA measurements in June-2005*			
	
	*R_50_*	*R_p_*	*E_0_*	*E_p,0_*	*R_50_*	*R_p_*	*E_0_*	*E_p,0_*
	*cm*		*MeV*		*cm*		*MeV*	
6 MeV	2.36	2.99	5.50	6.16	2.37	3.09	5.52	6.36
9 MeV	3.58	4.42	8.33	9.03	3.64	4.60	8.48	9.38
12 MeV	4.92	6.06	11.46	12.32	5.02	6.30	11.70	12.79
15 MeV	6.18	7.54	14.39	15.29	6.24	7.76	14.77	15.74
18 MeV	7.47	9.22	17.41	18.69	7.61	9.42	17.73	19.09
22 MeV	8.71	10.90	20.28	22.09	8.91	10.95	20.76	22.24

Evans *et al.* reported the efficacy of a commercially available energy monitor using multiple parallel plate chambers to find changes in electron energies.[10] They opined that subtle energy shifts in linacs occur due to a) bending magnet assembly and b) sensitivity of commercially available beam monitoring systems.[10] The design of their monitor chamber could indicate 11% change in dosimeter signal detecting 2 mm I_50_ variations in low-energy electron beams. For high-energy electron beams, around 20 MeV, a 2.5% change in signal could be detected for 2 mm I_50_ variations. In the light of the above work, we feel that our measured changes in electron energy ratios appear to be genuine and realistic. The beam profiles measured with RFA for these electron beams remained consistent when these small changes in electron energies were detected.

The simple QA procedures mentioned in this paper could be useful in all radiotherapy departments for studying beam stability. It is felt that one set of 20 cm thick perspex phantom could be used for at least 5 years, after which they may become fragile due to continuous irradiations. We are making efforts to participate in TLD postal dose inter-comparison with any nearby accredited radiological dosimetry laboratory to validate our beam level dosimetry.
